# Identifying Molecular Changes in *Giardia lamblia* Stages Using Hyperspectral Raman Microscopy

**DOI:** 10.3390/diagnostics15172161

**Published:** 2025-08-26

**Authors:** Felicia S. Manciu, Breanna C. Pence, Blessing A. Ibechenjo, Marian Manciu, Sudhir Bhattarai, Siddhartha Das

**Affiliations:** 1 Department of Physics, University of Texas at El Paso, El Paso, TX 79968, USA; baibechenjo@miners.utep.edu (B.A.I.); mmanciu@utep.edu (M.M.); sbhattarai3@miners.utep.edu (S.B.); 2 Border Biomedical Research Center, University of Texas at El Paso, El Paso, TX 79968, USA; sdas@utep.edu; 3 Department of Biological Sciences, University of Texas at El Paso, El Paso, TX 79968, USA; bcpence@miners.utep.edu

**Keywords:** Raman microscopy, *Giardia*, confocal fluorescence microscopy, trophozoites, cysts, giardiasis, non-tagged analytes

## Abstract

**Background/Objectives:** Giardiasis is one of the oldest-reported infectious diseases worldwide. It affects individuals with weakened immune systems and progresses into chronic infection if untreated. Morphological analysis and visualization of cell shapes using unlabeled or fluorophore-labeled samples are commonly employed to identify the parasite. **Methods:** To distinguish molecular content variations between trophozoites and infectious type I cysts, the current research presents an alternative approach based on label-free Raman microscopy. **Results:** Constituents responsible for plasma membrane thickening and cyst wall formation during encystation, such as N-acetylgalactosamine (GalNAc) and N-acetylglucosamine (GlcNAc) were detected. Although these two coexisting compounds have similar molecular structures, their spectroscopic distinction and visual localization through Raman microscopy are achievable. While immature and non-viable cysts contain a larger amount of GlcNAc, a potential transition of this moiety to GalNAc might occur as the cysts mature and become infectious. Other Raman results revealed changes in the oxidation states of heme-binding proteins and in lipid–protein metabolism, each serving as an additional protection mechanism that the parasite employs for survival. Complementary bright field and confocal fluorescence microscopy results corroborate the Raman outcomes. **Conclusions:** The molecular-level findings of this work, which presents a detailed spectroscopic analysis of *Giardia*’s encystation and excystation stages, substantiate the need to use complementary methods for monitoring the parasite’s dynamics and efficacy in terms of self-protection. This alternative method provides accurate insights for further understanding the multifaceted factors involved in *Giardia*’s encystation process and its acquired resistance to external stimuli.

## 1. Introduction

*Giardia lamblia*, a parasite discovered in 1681, continues to infect approximately 200 million people worldwide, with 500,000 new cases occurring each year [1,2]. Since infection in human hosts occurs when cysts are transmitted through contaminated water and food, the Food and Agriculture Organization of the United Nations (FAO)/WHO has ranked *Giardia* as the 11th most significant foodborne parasite globally [3]. Diarrhea, vomiting, dehydration, and malabsorption are typical symptoms of giardiasis. Nearly 33% of people have been infected at some point in their lives, with a higher rate of occurrence in the populations of developing countries [2], such as the 55.3% seropositivity rate reported in Mexico [4]. These percentages could be even higher, considering the presence of infection in asymptomatic individuals, meaning that the disease goes undiagnosed and underreported [5,6]. Infection occurs when the ingested cysts migrate from the stomach to the upper part of the duodenum, where, through excystation, trophozoites are released [7,8,9,10]. Long-term effects and illnesses, such as irritable bowel syndrome, fatigue syndrome, and food allergies, are consequences of untreated giardiasis. Individuals with weakened immune systems are especially prone to developing chronic infections [7,8,9,10]. Although not life-threatening, the reported resistance of giardiasis to standard treatment with nitroimidazole-based drugs and the mutation of the organism are of worldwide concern [11,12,13,14,15,16,17], and have boosted new research into drug development [18].

Microscopic visual examination of cysts or trophozoites in stool samples (ova and parasite examination) and immunological methods are regularly employed for the detection of protozoa [19]. Additionally, fluorescence microscopic analysis is essential to confirm the presence of *Giardia* in test samples. In immunological testing, specific antibodies are required. Sometimes, these tagging substances react with targets other than the parasite of interest, resulting in false-positive outcomes and unnecessary additional tests [20]. Consequently, immunological tests do not provide accurate differentiation, especially between viable (infective) protozoan cysts and non-viable ones. Since, frequently, a mixture of type I (oval-shaped, viable, and excystation-efficient) and type II (round-shaped, non-viable, and excystation-inefficient) cysts exists in vivo and in vitro [21,22,23], finding alternative methods of investigation of these anaerobic protozoa to complement the morphological ones is essential. These new investigation methods need to detect, with high accuracy, the processes involved in *Giardia*’s dynamics at the molecular level and reveal the pathogen’s mechanisms of infection.

An alternative method for such analysis proposed herein is Raman microscopy, which has contributed to the diagnosis of non-life-threatening and terminal diseases. This non-invasive imaging technique combines standard Raman spectroscopic results with optical imaging, allowing for fast in situ measurements of non-tagged moieties. Raman microscopy has been used to identify structural content variations in different types of bacteria, viruses, eukaryotic cells, and cancer, with high accuracy [24,25,26,27,28,29,30,31]. Besides benefiting from an insensitivity to water absorption, which can impede investigations of biological analytes, it provides well-defined vibrational features. This latter capability is advantageous compared with the commonly broad bands obtained in photoluminescence analysis. Recording thousands of Raman spectra in a few minutes, with a single spectrum recorded in 50 ms or less, is another valuable advantage. Not only does each Raman spectrum give multiple features for analysis through the integrated intensities or areas of the relevant peaks, but the large number of spectra obtained through rapidly repeated sampling and the simultaneous detection of all structural biomarkers obtained with confocal Raman microscopy can uniquely resolve and discriminate among the existing variations. This improved signal collection process, without additional labeling requirements by external agents and dyes, enables accurate analysis. It is a significant advantage for the fast, direct identification of molecular changes in cells’ biochemical compositions. One drawback of the future use of Raman microscopy in *Giardia* studies is that existing Raman experimental data are insufficient, making the technique less effective for understanding all the structural changes. While some molecular variations could be obvious, as in our recent study of drug effects on *Giardia* [24], such a limitation would become evident if some Raman peaks were to exhibit asynchronous or undetectable variations.

In this study, we use confocal Raman microscopy as a new modality for the label-free detection of molecular changes occurring in *Giardia lamblia* stages and for visually locating those regions in which such changes occur through high-resolution imaging. In addition to analyzing the biochemical composition of trophozoites, the factors and mechanisms responsible for structural changes in *Giardia* cysts will be surveyed and discussed. Since our previous results revealed modifications in the heme protein environment and transformations of iron from ferric to ferrous forms after drug treatment [24], special attention will be given to such protein activities. The genes of two family members of CPR (NADPH-cytochrome P450 oxidoreductase) and four members of the *cyt b5* family are present in *Giardia* [18]. The most prevalent forms are the *b* and *c* hemes, with the iron ion at the center of the porphyrin compound having different oxidation states. Iron coordination has been used to classify them and determine their contribution to biological functions and various processes [32,33,34]. We anticipate that some of these five heme-binding proteins may undergo modifications during encystation in this eukaryote that lacks mitochondria. Other moieties that are responsible for wall thickness increases are also expected to become more dominant. This work lays the foundation for the Raman spectroscopic detection of specific molecular changes occurring in *Giardia*’s final stages (i.e., trophozoites and cysts) in defined contexts and potentially fills interpretive gaps that are not covered by other methodologies. In the future, such results could provide a newer, faster, and more comprehensive method of detecting and examining this pathogen. This, in turn, could speed up the discovery of methods for effectively treating chronic giardiasis.

## 2. Materials and Methods

### 2.1. Sample Preparation

Standard procedures were followed for culturing the *Giardia lamblia* assemblage of trophozoites (strain WB C6; ATCC No. 30957) and generating cysts [21,22,23]. To culture the trophozoites, they were incubated in modified Diamond’s TYI-S-33 medium, supplemented with 5% heat-inactivated adult bovine bile, 0.5 mg/mL bovine bile, and 100 µg/mL piperacillin at 37 °C in screw-cap glass tubes until a confluence of 80–90% was reached [21]. The cultures were divided routinely every 3–4 days. To generate cysts in vitro, trophozoites were cultivated in T25 flasks to ~85% confluency. Two different procedures were followed, namely, the 72-hour protocol by Gillin et al. and the high-bile method [22,23]. The medium was replaced with a TYI-S-33 medium at a pH of 7.8, supplemented with 5% heat-inactivated adult bovine serum, lactic acid (5 mg/mL), and porcine bile (250 mg/mL) [22]. The parasites were left in this new medium for 72 h to allow for the complete generation of cysts. To generate cysts using the high-bile procedure, the medium for trophozoites in the T25 flasks was replaced with TYI-S33 supplemented with 10% adult bovine serum and 5 mg/mL bovine bile at pH 7.8 for 24 h [23]. In this case, following the Gillin et al. protocol resulted in mature cysts forming in the culture within 48 h. Mature cysts were obtained after 18 h with the high-bile method. A mixture of type I (oval-shaped and viable) and type II (round-shaped and non-viable) was usually produced [22,23]. The high-bile encystation protocol generated mostly type I cysts with better excystation efficiency. For both preparation methods, the cysts were collected by centrifugation (2500× *g* for 10 min at 4 °C), washed three times in cold double-distilled water, and kept in water overnight at 4 °C.

The samples for Raman analysis were fixed with 4% paraformaldehyde solution (Sigma-Aldrich, St. Louis, MO, USA) for 10 min, followed by washing in PBS and double-distilled water several times. Samples of approximately 1.0 × 10^4^ cells per 30 µL volume were drop-cast onto coverslips and allowed to air-dry at room temperature. For fluorescent confocal microscopy analysis, the cysts were blocked with 5% normal goat serum at room temperature for 20 min, then labeled with 1:100 anti-*Giardia* (cyst) antibody sc-57744 (Santa Cruz Biotechnology, Dallas, TX, USA) and diluted in 1% normal goat serum overnight at 4 °C. The samples were then washed three times with PBS and incubated in 1:400 donkey anti-mouse secondary antibody, conjugated to Alexa 568 A10037 (Invitrogen, Waltham, MA, USA) for 1 h at room temperature. Each sample was also incubated in 50 µL of 1 µg/mL DAPI solution at room temperature for 15 min after washing it three times in PBS. Before their analysis with a confocal microscope (Carl Zeiss Laser Scanning Systems LSM 700, Oberkochen, Germany), the samples were washed three more times with PBS and mounted on glass slides with Vectashield Antifade Mounting Medium (Vector Laboratories, Newark, CA, USA).

### 2.2. Instruments

The measurements of surface Raman mapping images of the trophozoites and cysts were carried out with an alpha 300RAS WITec confocal Raman system (WITec GmbH, Ulm, Germany) equipped with a microscope and coupled to a triple grating monochromator/spectrograph via an optical fiber of 50 μm in core diameter. The 532 nm light excitation of the frequency-doubled neodymium-doped yttrium–aluminum–garnet (Nd:YAG) laser was kept at about 3 mW of power output to avoid sample photodegradation. A 100X air objective lens (Nikon, Tokyo, Japan) of 0.90 numerical aperture (NA), a thermoelectrically cooled Marconi CCD camera, and a 600 lines/mm grating providing a spectral resolution of about 2 cm^−1^ were used for data acquisition. A piezoelectric stage, which is software-controlled, was employed for sample scanning and fast spectral recording using an integration time of 50 ms per spectrum. Arrays of 100 × 100 Raman spectra were collected for surface Raman mapping; 50 μm × 50 μm scan sizes were used for multiple-cell images, and 20 μm × 20 μm scan sizes were used for single-cell images.

A Carl Zeiss LSM 700 (Zeiss, Oberkochen, Germany), equipped with a 63X oil Plan-Apochromat Zeiss objective lens and two different laser excitations of 568 nm (set to level 5 and gain 946) and 405 nm (set to level 10 and gain 930), was used to perform confocal microscopy analysis of fluorescently labeled *Giardia* samples. Both image capture and analysis were performed with the Zen 2009 software (Zeiss, Oberkochen, Germany).

## 3. Results and Discussion

**Figure 1:**
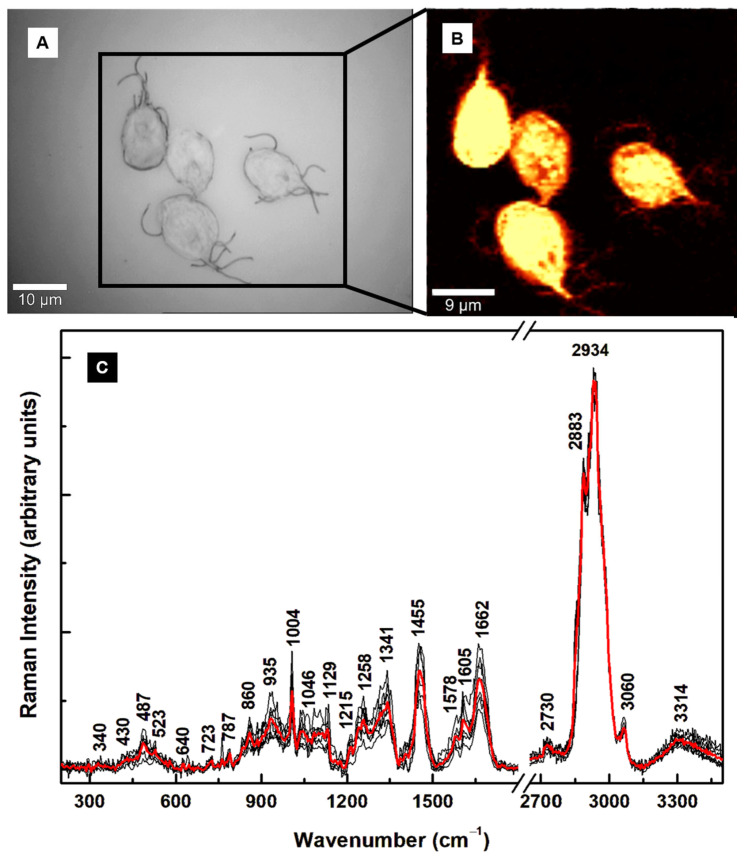
(**A**,**B**) Representative optical image and the corresponding surface Raman mapping image of multiple trophozoites. A bright yellow pseudo-color corresponds to a higher Raman intensity. (**C**) Integrated Raman spectra associated with several confocal Raman images (black lines) and the average of these integrated spectra (red line).

*Giardia lamblia* stages have been investigated extensively using confocal fluorescence microscopy and TEM [7,8], but far fewer studies have used Raman spectroscopy [24,28,35]. Before ascertaining the cysts’ characteristic spectroscopic signatures and identifying the infectious cysts, the first step was to determine the trophozoites’ specific vibrational lines using Raman spectroscopy. These results are presented in Figure 1A–C. An optical image (Figure 1A) and a representative confocal Raman image recorded with hyperspectral resolution (Figure 1B) are shown in this figure, in addition to the trophozoites’ spectra in Figure 1C. Each of the black line spectra in Figure 1C is an average of 10,000 independent spectra acquired during the Raman surface mapping with the laser. The red Raman spectrum is the average of all the integrated black-line Raman spectra. Since the bio-structural composition of the cells might vary from cell to cell, using these spectral averages for images containing more than one cell accounts for the intrinsic non-uniformity of any sample.

Appropriate background subtraction, normalization to the most intense line at 2934 cm^−1^, and elimination of the featureless, silent Raman region from 1800 to 2650 cm^−1^ were performed. The multitudes of vibrational lines seen in Figure 1C are summarized, along with their tentative assignments, in Table 1. From all these vibrational lines, some weak features in the low- and mid-frequency regions at 340, 430, 487, 523, 640, and 1341 cm^−1^ (more intense) are associated with the heme proteins [25,26,28,32,33]. Although *Giardia* lacks mitochondria, it does contain rudimentary organelles, such as mitosomes or mitochondrion-like organelles (MLO) [8]. These organelles, which replicate through mitosis [36], contain iron–sulfur (Fe–S) moieties with important roles in assembling proteins in the absence of ATP production (usually generated by oxidative phosphorylation). Phenylalanine, amides, glycogen, tryptophan, guanine, adenine, and phospholipids dominate in the mid-frequency region. The strongest Raman lines are in the high-frequency region and are attributed to lipids and proteins [25,28].

**Figure 2:**
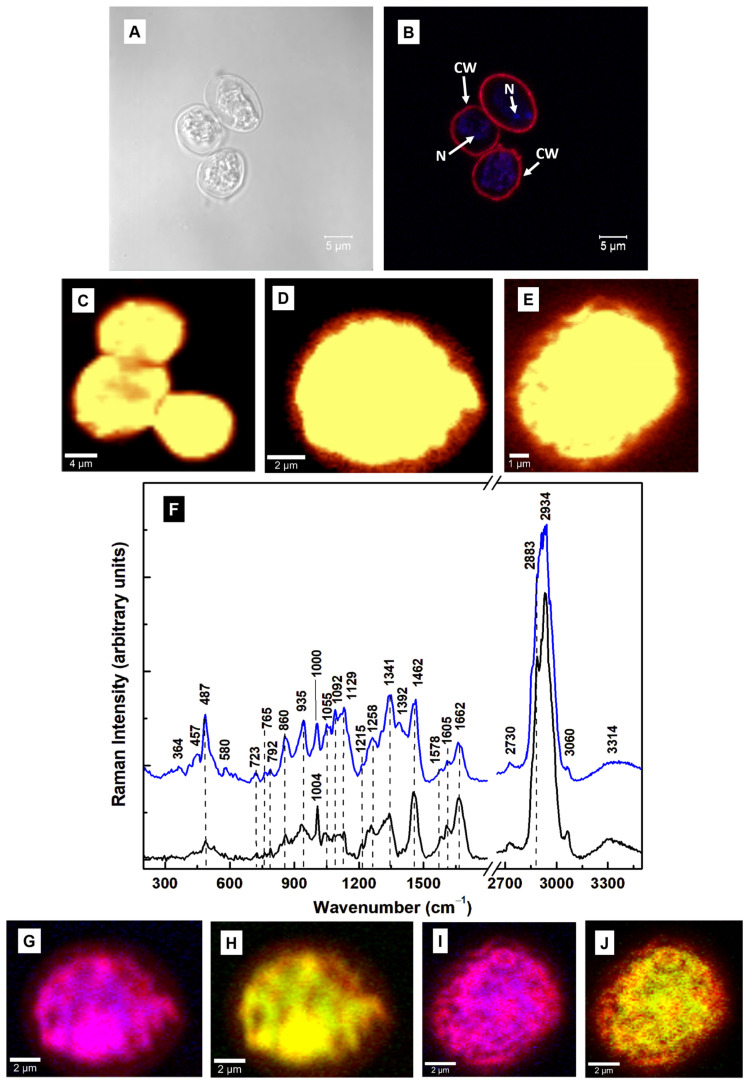
(**A**) Bright-field image of cysts that were generated using the Gillin et al. method [22]. (**B**) Confocal fluorescence image taken after labeling the cysts with anti-*Giardia* (cyst) antibody sc-57744 for cyst walls (red) and with DAPI for nuclei (blue). (**C**–**E**) Representative confocal Raman images of multiple and independent oval-shaped cysts. A bright yellow pseudo-color corresponds to a higher Raman intensity. (**F**) The overall average (blue line) of the integrated Raman spectra associated with images (**C**–**E**) and the spectrum of trophozoites (black line), which is plotted for reference. These two spectra are translated vertically for easier visualization. (**G**–**J**) Confocal Raman mapping images of the cysts in images (**D**,**E**), performed by selectively filtering the vibrational lines at 487 cm^−1^ (blue pseudo-color) and 1341 cm^−1^ (green pseudo-color). A red pseudo-color was used for the body of the cell. Thus, the magenta color in images (**G**,**I**) and the yellow color in images (**H**,**J**) are formed by an overlap between red and blue or red and green, respectively. **Legend:** CW: cyst wall and N: nucleus.

In vitro cultures contain a mixture of type I and type II cysts, with the majority of the former or the latter, depending on the sample preparation. Therefore, to identify the Raman signature of the infectious type I, we selectively mapped multiple and single oval-shaped cysts, which were prepared using the Gillin et al. method [22]. The bright field and confocal fluorescence microscopy images of the cysts are shown in Figure 2A and Figure 2B, respectively. The overall cell morphology can be investigated by the fluorescent labeling of the cyst wall with anti-cyst antibody and of the nucleic acids with DAPI. The Raman images and the average of their integrated spectra (blue line) are presented in Figure 2C–F. For easier comparison of the vibrational differences between cysts and trophozoites, the average of the integrated Raman spectra of trophozoites (black line) is also plotted in Figure 2F. These spectra were subject to background subtraction, vertical translation, and normalization to the most intense Raman vibrational line at 2934 cm^−1^.

Different activities with increased intensities for the Raman vibrations seen at 487, 860, 935, and 1341 cm^−1^ were observed in the integrated spectrum of the cysts compared to that of the trophozoites. The 457 and 487 cm^−1^ vibrational lines may be associated with Fe–CO and methionine stretching [25,35], implying structural modifications in the heme-binding and wall proteins. *Giardia* lacks the encoding enzymes required for heme biosynthesis but contains several genes that encode putative heme-binding proteins. During encystation and the formation of protective cyst walls, large quantities of cyst wall proteins (CWPs) are synthesized and transported by encystation-specific vesicles (ESVs) from the endoplasmic reticulum (ER) to the cell surface. Methionine is an essential moiety in the biosynthesis of the phospholipids and proteins found in the cell wall material. Others are leucine, serine, valine, and tryptophan.

To understand more about the molecular constituents’ contributions and their distribution within the cell, selective imaging was performed in Figure 2G–J by filtering to map the 487 cm^−1^ (blue pseudo-color) and 1341 cm^−1^ (green pseudo-color) vibrations. In these images, a red pseudo-color was used for the overall body of the cell. Thus, the magenta color in the (2G) and (2I) images represents the overlap of red and blue, and the yellow color in images (2H) and (2J) represents the overlap of red and green, respectively. The striking similarity of the blue and magenta locations with those of green and yellow suggests that both features at 487 and 1341 cm^−1^ correspond to the same analyte. We tentatively assigned them to N-acetylgalactosamine (GalNAc), which is an important component of *Giardia*’s cyst wall, or the O–S–O vibration of oxidized sulfur compounds and hydrogen sulfide species [37,38,39]. The potential association of these lines with GalNAc was suggested by a comparison with their presence in the reported spectrum of the compound by Ashton et al. [37]. Noticeable intensity increases are observed for the vibrations at 1055, 1092, 1129, and 1215 cm^−1^, which could originate from GalNAc and oxidized sulfur species. The former features could also be attributed to sphingomyelin structural changes and the symmetric ring breathing of C–C and C–O–C glycosidic links [35,40]. Sphingomyelin is one of the major sphingolipids found in *Giardia*, with abundant modifications during encystation [40]. Other structural differences between cysts and trophozoites are indicated by a relative decrease, broadening, and slight shift to 1000 cm^−1^ in the cysts’ phenylalanine line at 1004 cm^−1^, and comparatively diminished lipid, phospholipid, and protein contents, as indicated by the peaks at 1258, 1578, 1605, 1662, and 2883 cm^−1^ in the cyst spectrum.

**Figure 3:**
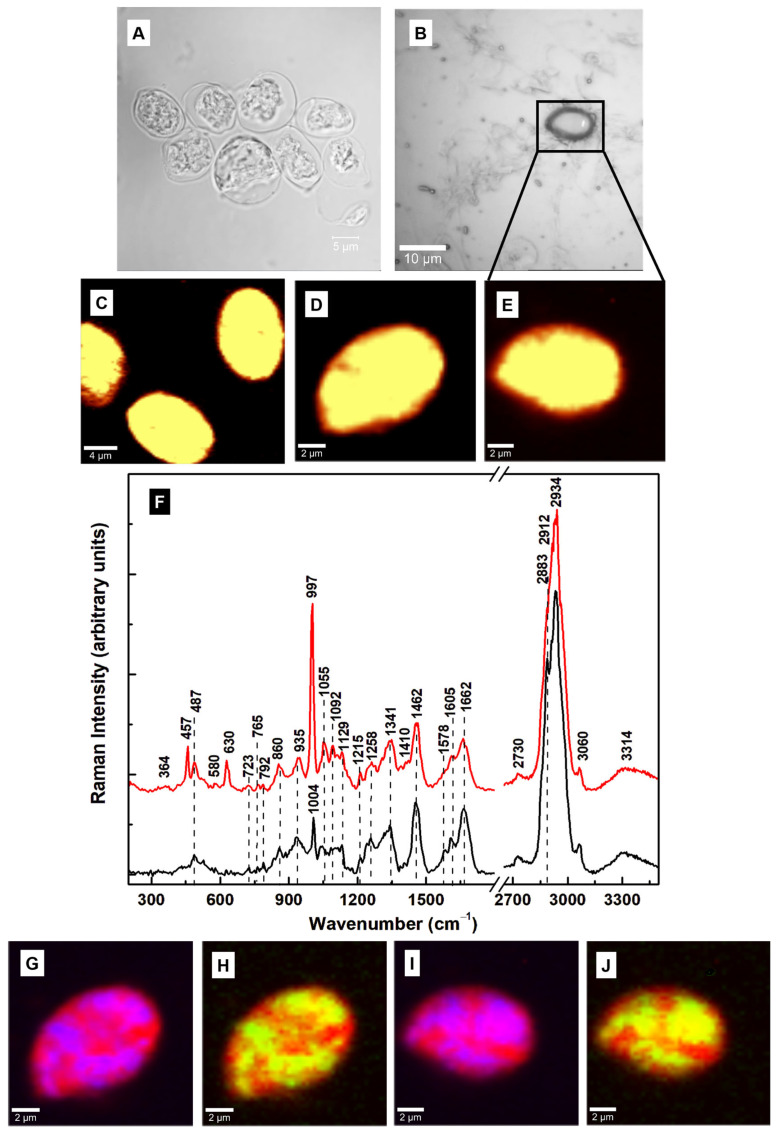
(**A**) Bright field image of cysts that were generated using the Gillin et al. method [22]. (**B**) Optical image showing where surface confocal Raman mapping was performed. (**C**–**E**) Representative confocal Raman images of multiple and independent oval-shaped cysts. A bright yellow pseudo-color corresponds to a higher Raman intensity. (**F**) The overall average (red line) of the integrated Raman spectra associated with images (**C**–**E**) and the spectrum of trophozoites (black line), which is plotted for reference. These two spectra are translated vertically for easier visualization. (**G**–**J**) Confocal Raman mapping images of the cysts in images (**D**,**E**), performed by selectively filtering the vibrational lines at 997 cm^−1^ (blue pseudo-color) and 630 cm^−1^ (green pseudo-color). A red pseudo-color was used for the body of the cell. Thus, the magenta color in images (**G**,**I**) and the yellow color in images (**H**,**J**) are the result of an overlap between red and blue or red and green, respectively.

Further Raman microscopic investigations of the cysts, prepared by the same method [22] but taken from another batch of samples, are presented in Figure 3A–J. Figure 3A,B shows the associated brightfield and optical images. The small rectangle in Figure 3B shows the location where the confocal Raman image in Figure 3E was obtained. The integrated Raman spectra of the cysts (red line) and trophozoites (black line) are plotted in Figure 3F. In this case, of note in the integrated Raman spectrum of the cysts are the appearances of three new peaks at 457, 630, and 997 cm^−1^. While the first two vibrational lines correspond to Fe–CO and methionine stretching, respectively, the latter intense line is near the phenylalanine peak at 1004 cm^−1^ [25,35]. The appearance of a strong vibrational feature at 997 ± 3 cm^−1^ was also observed in our previous study of trophozoites treated with oseltamivir [24], a common drug for alleviating the symptoms of seasonal influenza. It was attributed tentatively to an altered downregulation of the non-heme iron-containing monooxygenase PAH (essential for catalyzing the oxidation of phenylalanine to tyrosine). Another observation was the absence of the extracellular vesicle release of oseltamivir-treated trophozoites and the tendency of the cells to undergo encystation [24]. The most intense peak, observed at 997 cm^−1^, could also be assigned to deoxyribonucleic acid (DNA) or some modified RNA [28], supporting our previous remarks regarding GalNAc’s role in the compositional modification of nucleic acid structures. Although cysts contain multiple nuclei, this strong increase in the intensity of this feature for non-viable, non-mature, and possibly mutated cysts is unusual. Because of its relationship to phenylalanine, downregulated phenylalanine hydroxylase (PAH) activity is a more likely mechanism.

As for the oval-shaped type I cysts investigated previously (Figure 2), we selectively imaged the locations of the most dominant vibrational changes, namely, the lines at 997 cm^−1^ (blue pseudo-color) and 630 cm^−1^ (green pseudo-color), with red pseudo-color for the overall cell body. These images are shown in Figure 3G–J. The magenta color is formed by the overlap of blue (997 cm^−1^) and red (cell body), and the yellow by the overlap of green (630 cm^−1^) and red. Although the 630 and 997 cm^−1^ lines differ from those at 487 and 1341 cm^−1^, the similarities in their locations in the cyst body are intriguing. Once more, the resemblance in their locations is not only indicative that they belong to the same analyte but also that this moiety has a chemical conformation similar to that detected for the cysts in Figure 2. A tentative identity for this analyte is N-acetylglucosamine (GlcNAc), another important component of *Giardia*’s cyst wall, with a structure related to that of GalNAc [37,41]. Even though GalNAc originates from galactose and GlcNAc from glucose, their structure differs only by a hydrogen atom substituting an acetylated amino group at a carbon position in the sugar ring. At this position in the compound, GalNAc has a carbon-axial hydroxyl group, while GlcNAc has an equatorial hydroxyl group. This subtle structural difference, which is evident in the current Raman results, affects the glycosylation process and the resulting cyst structure, leading to distinct biological functions and interactions. For example, when they are attached to the hydroxyl groups of serine or threonine residues, respectively, on proteins, both GalNAc and GlcNAc could be involved in O-linked glycosylation. Also worth mentioning is GalNAc’s role in delivering therapeutic nucleic acids, such as siRNAs. Again, increases in the intensities of the vibrations at 1055, 1092, 1129, and 1215 cm^−1^ were observed in the spectrum of the cysts relative to those for trophozoites, along with weakening in the vibrations of lipids, phospholipids, and proteins at 1258, 1341, 1462, 1578, 1605, and 1662 cm^−1^. Additional structural variations of lipid content are seen in the high-frequency region at 2883 and 2912 cm^−1^.

Although the cysts imaged in Figure 3C–E have oval shapes, a closer look at the bright-field image in Figure 3A reveals non-lysed or residual trophozoites and a couple of cysts with round shapes. This observation, together with the previous findings concerning the 630 and 997 cm^−1^ peaks, indicates incomplete encystation. Furthermore, since the Raman spectra of both GlcNAc and GalNAc contain vibrations at 1055, 1092, and 1129 cm^−1^, a dynamical biochemical transformation between the two compounds may take place during the encystation process and the cysts’ maturation [17]. A more intense 1129 cm^−1^ line has been reported as being characteristic of GalNAc [37]. This Raman vibration can be considered a potential signature for type I mature cysts. Since the intensity of this line is stronger in the spectrum of the cysts in Figure 2F, showing type I cysts, than in Figure 3F, showing immature cysts, this substantiates the biochemical change. Additionally, the 630 cm^−1^ feature, which is present in the reported spectrum of GlcNAc but is absent in that of GalNAc [37], does not exist in the Raman spectrum of type I cysts (Figure 2).

**Figure 4:**
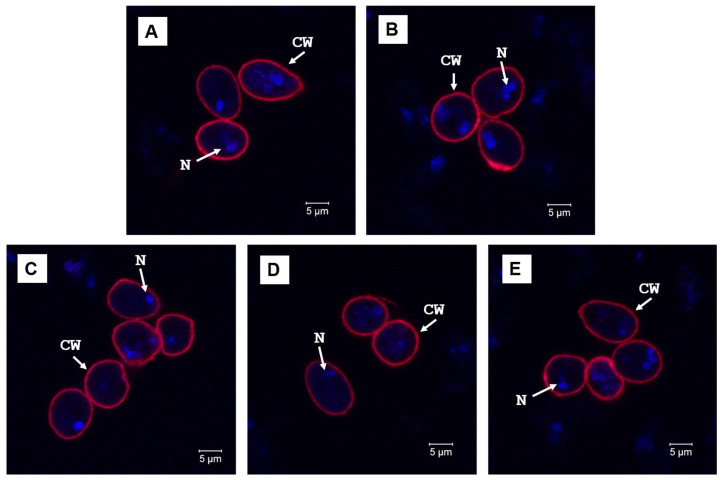
(**A**–**E**) Confocal fluorescent microscopy of *Giardia* cysts after labeling with anti-*Giardia* (cyst) antibody sc-57744 for cyst walls (red) and with DAPI for nuclei (blue). The (**A**,**B**) panels show selective imaging of oval (type I) and immature cysts. (**C**–**E**) Imaging of multiple cysts, revealing unformed cysts and uncontained nuclei. **Legend:** CW: cyst wall and N: nucleus.

Supporting evidence using the fluorescence microscopy of these cysts is presented in Figure 4A,E. While the cyst wall (red labeling) looks similar in images (A) and (B) of the selective representations of type I and immature cysts, respectively, there are large amounts of uncontained nucleic acids with invisible cyst walls that are associated with the latter (image (B)). Slightly thicker walls can be observed for type I cysts, which contain more GalNAc, in agreement with our previous suggestion of a potential conversion between GlcNAc and GalNAc during the encystation and maturation of the cysts. Additional evidence of mixtures of these two types of cysts is presented in Figure 4C–E, with a preponderance of unformed cysts and disordered nuclei.

**Figure 5:**
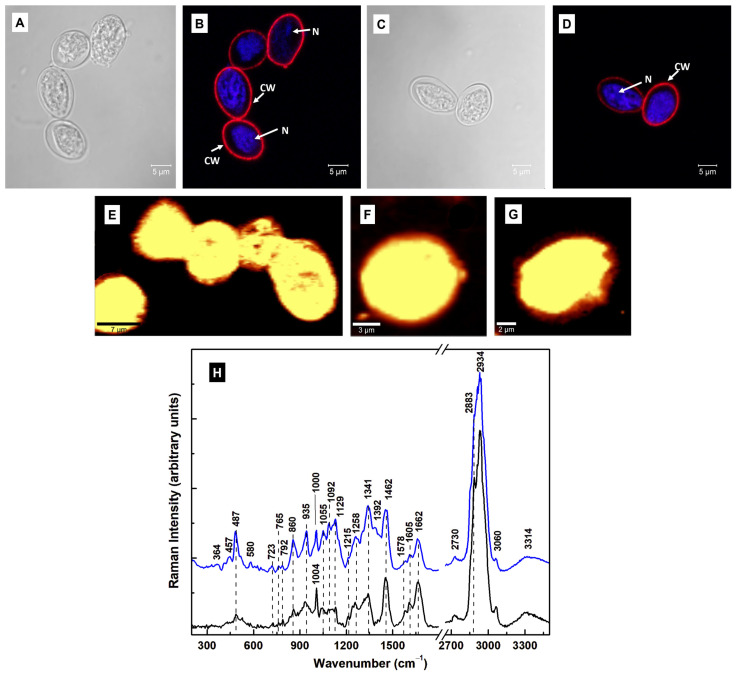
(**A**–**D**) Bright-field images and the associated confocal fluorescence images of *Giardia* cysts generated using the high-bile method [23] and after labeling with anti-*Giardia* (cyst) antibody sc-57744 for cyst walls (red) and with DAPI for nuclei (blue). (**E**–**G**) Confocal Raman images of multiple and single cysts. (**H**) The overall average (blue line) of the integrated Raman spectra associated with images (**E**–**G**) and the spectrum of trophozoites (black line), which is plotted for reference. **Legend:** CW: cyst wall and N: nucleus.

The bright field, confocal fluorescence, and Raman images of viable type I cysts that were generated using the high-bile procedure [23] are presented in Figure 5A–G. A uniform distribution of oval-shaped cysts with well-defined and slightly thicker walls can be observed in Figure 5A–D, confirming that the high-bile protocol generates more type I cysts, as previously reported [23]. The average (blue line) of the integrated Raman spectra of multiple- and single-cyst images and that of the trophozoites are shown in Figure 5H. The Raman spectrum of the cysts resembles the one obtained in Figure 2.

**Figure 6:**
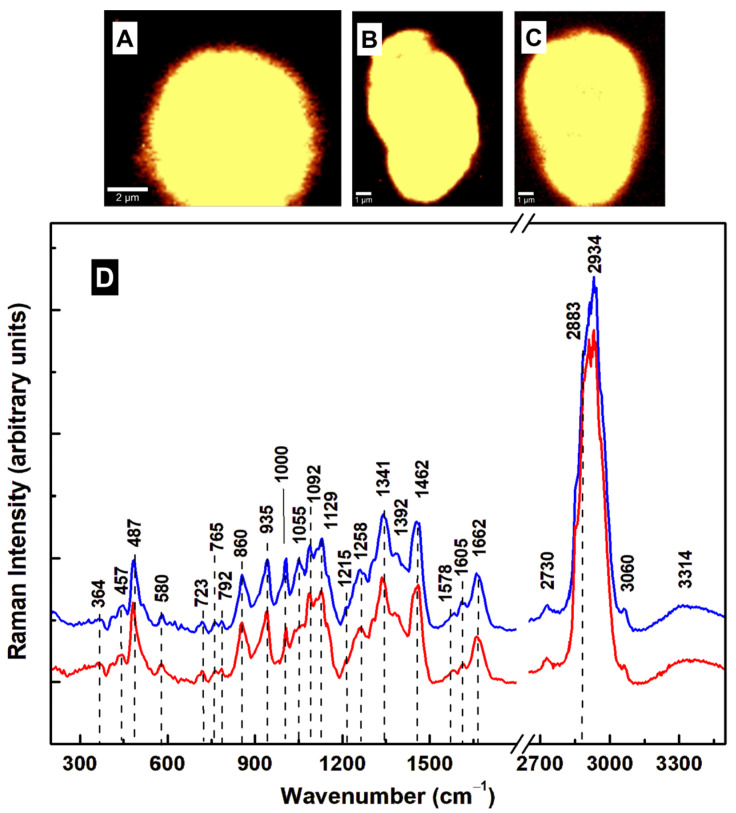
(**A**–**C**) Representative confocal Raman images of round-shaped and oval-shaped cysts. (**D**) Integrated Raman spectra associated with round-shaped (red line) and oval-shaped (blue line) cysts, which have been translated vertically for easier visualization and comparison.

The comparative Raman microscopic investigation of type II cysts (round-shaped and red line spectrum) and type I cysts (oval-shaped and blue line spectrum) is presented in Figure 6A–D. The integrated Raman spectra look almost identical for both cyst types. However, there are some small differences, such as the intensity increase (from red to blue) in the vibrational lines at 1000 and 1055 cm^−1^. While the former is close to the phenylalanine line at 1004 cm^−1^, the latter is characteristic of GalNAc [37]. Only a shoulder can be observed in the spectrum of type II cysts at 1000 cm^−1^. Although phenylalanine is not directly involved in O-linked glycosylation, it has a hydrophobic benzyl side chain. Thus, indirectly, some of its residues may influence the local protein structure and the efficiency of O-linked glycosylation, especially when interacting with serine or threonine. If the hexosamine biosynthetic pathway (HBP) among all other amino acid and lipid metabolisms is considered, the amino acids resulting from protein breakdown could enter carbohydrate metabolism pathways (i.e., pyruvate, acetyl-CoA, etc.) [17,39], potentially leading to GlcNAc or GalNAc synthesis.

**Figure 7:**
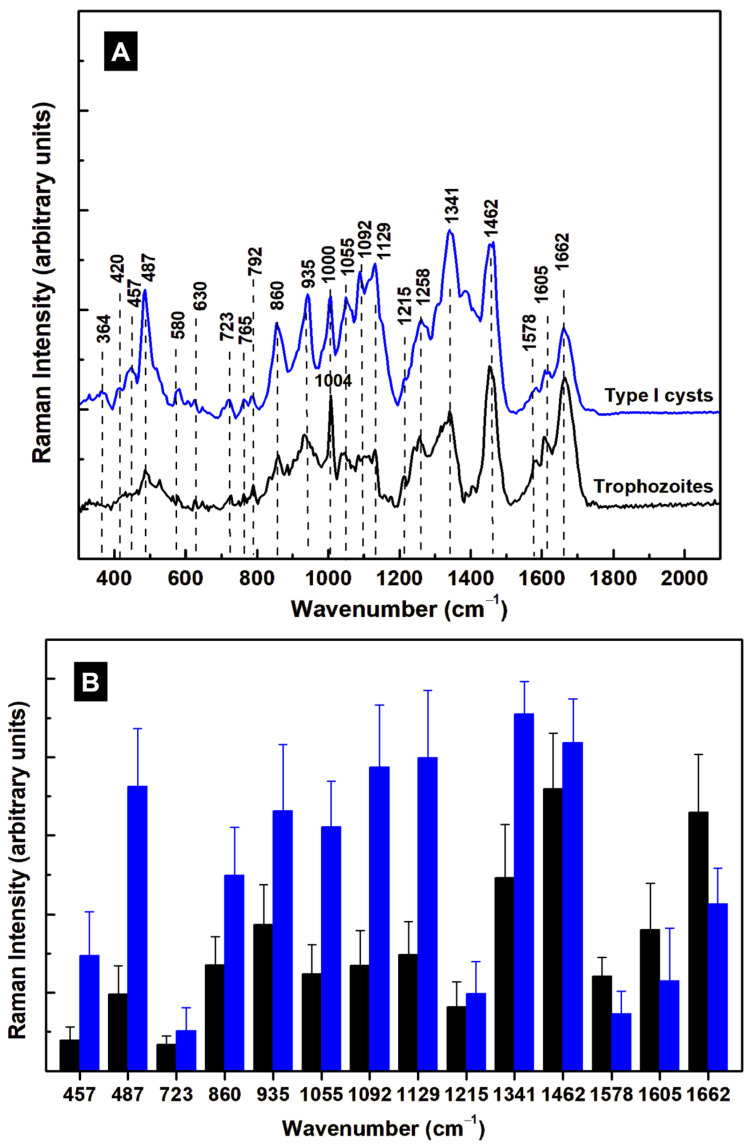
(**A**) Averages of the integrated Raman spectra of trophozoites (black line) and type I cysts (blue line), as labeled. (**B**) Maximum intensity of specific Raman vibrations, demonstrating structural changes during encystation.

For easier comparison of the similarities and differences between type I (infectious) cysts and trophozoites, Figure 7A presents the averages of their integrated Raman spectra. We have kept the same color-coding: black for trophozoites and blue for type I cysts. Vibrational changes in the low-frequency region of 300–500 cm^−1^ can be attributed to modifications of hemeprotein conformations, such as iron oxidation states (ferrous or ferric states) or iron proximal ligands (Fe–CO and Fe–S bonds) [32,35], as previously mentioned in the analysis of trophozoites. For example, the 457 cm^−1^ line (Fe^II^–CO stretching mode) of trophozoites shows an increase in intensity for type I cysts. The appearance of other weak vibrations at 364, 420, 580, and 1390 cm^−1^ in the cyst spectrum supports the idea of an interchange between Fe^II^ and Fe^III^ [32,35].

More obvious modifications remain for the 487 cm^−1^ line and, in the mid-infrared region, at the 1055, 1092, 1129, and 1341 cm^−1^ bands, with all becoming much stronger for type I cysts. In addition to the major components, GalNAc and GlcNAc, in the formation of cyst walls with vibrations at 630, 1055, 1092, 1129, and 1215 cm^−1^, there is a weak Raman line at 723 cm^−1^ in the spectra of both trophozoites and cysts that can be assigned to sphingomyelin [39]. Since this compound has vibrations at 1068 and 1129 cm^−1^, the features recorded at 1055, 1092, and 1129 cm^−1^ could also indicate its presence. Many factors play significant roles in the complexity of *Giardia*’s encystation process. To increase the parasite’s chance of survival, while the ESVs help transport substances to the surface to strengthen the cell walls, some other substances, such as heme proteins and cholesterol, are modified or depleted.

Although the majority of lines display intensity increases in the spectrum of cysts, some show decreases with encystation, such as at 1578, 1605, and 1662 cm^−1^, which are associated with amide II and I, tryptophan, tyrosine, and phenylalanine. The variations in the intensity of these lines are represented with appropriate error bars in Figure 7B. A one-way MANOVA (multivariate analysis of variance) was employed to statistically identify the presence of Raman vibrations in trophozoites versus cysts. For two groups, this is equivalent to Hotteling’s T-squared test. Multicomparison corrections for individual *p*-values have been accounted for through Tukey–Kramer HSD tests. The fourteen measured relative heights of vibrational lines are considered dependent variables for two groups of data, namely, those of trophozoites and cysts. The statistical significance obtained for each variable is presented in Table 2.

Ten out of the fourteen relative heights of the Raman peaks at 457, 487, 860, 935, 1055, 1092, 1129, 1341, 1578, and 1662 cm^−1^ are significantly different between trophozoites and cysts, and can be used to identify the stages of individual cells.

## 4. Conclusions

Giardiasis, one of the oldest-reported infectious diseases worldwide [11,12,13,14,15,16], affects individuals with weakened immune systems. It can progress to chronic infection if left untreated [7,8,9,10]. The current research aims to understand the structural differences between trophozoites and viable, infectious cysts at a molecular level. These outcomes will enable accurate detection of the dormant causes of the infection, as well as the future development of drugs. While in vitro preparation methods generate mixtures of type I (oval-shaped, viable) and type II (round-shaped, non-viable) cysts [21,22,23], a preponderance of type I cysts has been reported for in vivo cysts. Morphological analysis and the shape visualization of unlabeled and fluorescently labeled samples are standard methods currently used to identify the parasite. Herein, we present a more accurate approach using a label-free alternative based on Raman microscopy, which has been used to distinguish structural content variations in various types of bacteria, viruses, eukaryotic cells, and cancer with high accuracy [24,25,26,27,28,29,30,31]. This alternative method provides insights into understanding the multifaceted factors involved in *Giardia*’s encystation process and its acquired resistance to external stimuli.

In addition to differentiating between the trophozoites and cysts, the current results reveal structural differences between the cysts themselves, such as immature cysts and non-viable (round-shaped) ones. For example, an expected outcome was identifying the constituents responsible for plasma membrane thickening and cyst wall formation during encystation, which is the primary mechanism by which the parasite protects itself from a harsh environment. The Raman results presented herein enable the distinction between the constituents GlcNAc and GalNAc. While both moieties coexist, a larger amount of GalNAc was observed in type I mature cysts. Even though these two compounds have similar molecular structures, using this method along with visual localization through Raman microscopy unlocks the detection of such variations. Another example is the observed changes in the oxidation states of heme proteins [32], an additional protection mechanism that the parasite employs for survival, which was seen when samples were treated with drugs [24]. The presence of sphingomyelin and changes in the vibrations corresponding to lipids, phospholipids, and proteins were also detected and analyzed.

This work, which is the first detailed spectroscopic analysis of *Giardia*’s stages (i.e., trophozoites and cysts), establishes that Raman microscopy is a valuable tool in accurately identifying the factors contributing to the parasite’s protection mechanisms. The outcomes, which cannot be obtained by standard morphological or confocal fluorescence microscopy, where the cyst shapes and wall thicknesses rely on individual interpretation, demonstrate that Raman microscopy is a step forward in finding a faster and more precise detection approach. It substantiates the need to use alternative, combinatorial methods of investigation to unlock new findings in protozoa’s protection efficacies and in determining the best tactics for overcoming giardiasis. Although not implemented in clinical practice or standard biological investigations, Raman microscopy could help tailor prophylactic and therapeutic strategies for personalized diagnostic and preventive treatment, particularly for high-risk populations.

## Figures and Tables

**Table 1:** Raman vibrational bands and their assignments with tentative attributions.

Raman Shift (cm^–1^)	Assignment	Attribution
340	C–C–C pyrrole ring deformation	Heme-binding proteins, cytochrome b_5_ [25,26,32,35]
430	C–C–C pyrrole ring deformation	Heme-binding proteins [25,26,32,35]
487	Fe–CO, C–C stretching	Heme-binding proteins, glycogen [28,32,35]
523	Fe–CO, S–S stretching	Heme-binding proteins, cysteine [32,35]
640	C–N, C–C twisting	Heme-binding proteins, guanine, tyrosine [25,28,32,35]
723	C–N ring breathing	Phospholipid, adenine [25,28,32]
787	Pyrrole breathing	Cytosine, nucleic acids [25,26,28,32,35]
860	C–C stretching, –CH out of plane bending, C–N bending	Tryptophan, glycogen, polysaccharide, arginine [25,28]
935	–CH_3_ symmetric stretching, N–C–N symmetric stretching, C–N bending	Lipids, proteins, arginine [25,28]
1004	Benzene ring breathing	Phenylalanine, collagen [25]
1046	C–C, C–N, C–O, O–S–O stretching	Lipids, proteins, glycogen, oxidized sulfur species [25,37]
1129	C–C, O–S–O stretching	Lipids, oxidized sulfur species [25,37]
1215	N–H bending, C–N stretching, O–S–O	Amide III, oxidized sulfur species [25,28,37]
1258	–CH_2_ deformation, N–H bending, C–N stretching	Lipids, Amide III [25,28]
1341	C–N breathing, –CH_2_ deformation	Heme proteins, lipids, adenine, guanine [25,26,32,35]
1455	C–N bending, –CH_3_ out-of-plane deformation	Lipids, proteins [25]
1578	N–H bending, C–N stretching	Tryptophan, Amide II, NADH [25]
1605	C–O stretching, C=C bending	Phenylalanine, tyrosine [25]
1662	C=O stretching, out-of-plane C–N stretching	Amide I [25]
1732	C=O stretching	Lipids, phospholipids, triacylglycerides [25]
2730	–CH stretching	Saturated fatty acids [25]
2883	–CH_2_ antisymmetric stretching	Lipids, raft-like ordered domains [25,28,33]
2912	–CH_3_ symmetric stretching	Lipids, proteins [25,28]
2934	–CH_3_	Proteins, fat, cholesterol [25,28]
3060	=CH	Unsaturated fatty acids [25,28]
3314	–NH, –OH	Proteins, water band [25,28]

Refs: [25,26,28,32,33,35,37].

**Table 2:** Raman vibrational bands and their statistical significance.

Raman Shift (cm^–1^)	*p*-Value
457	1.0 × 10^−3^
487	1.4 × 10^−5^
723	0.14
860	1.5 × 10^−3^
935	6.0 × 10^−3^
1055	1.4 × 10^−4^
1092	1.3 × 10^−5^
1129	1.8 × 10^−5^
1215	0.59
1341	2.3 × 10^−3^
1462	0.29
1578	1.8 × 10^−2^
1605	0.16
1662	3.7 × 10^−2^

## Data Availability

Most of the data generated or analyzed are included in the article. The remaining datasets used and/or analyzed during the current study are available from the corresponding author upon request.
